# Prebiotic Potential of Oligosaccharides Obtained by Acid Hydrolysis of α-(1→3)-Glucan from *Laetiporus sulphureus*: A Pilot Study

**DOI:** 10.3390/molecules25235542

**Published:** 2020-11-26

**Authors:** Adrian Wiater, Adam Waśko, Paulina Adamczyk, Klaudia Gustaw, Małgorzata Pleszczyńska, Kamila Wlizło, Marcin Skowronek, Michał Tomczyk, Janusz Szczodrak

**Affiliations:** 1 Department of Industrial and Environmental Microbiology, Institute of Biological Science, Maria Curie-Skłodowska University, Akademicka 19, 20-033 Lublin, Poland; paulinapolak2501@gmail.com (P.A.); m.pleszczynska@poczta.umcs.lublin.pl (M.P.); kamila.wlizlo@poczta.umcs.lublin.pl (K.W.); szczo@poczta.umcs.lublin.pl (J.S.); 2 Department of Biotechnology, Human Nutrition and Food Commodity Science, University of Life Sciences in Lublin, Skromna 8, 20-704 Lublin, Poland; kowalikklaudia2105@gmail.com; 3 Laboratory of Biocontrol, Application and Production of EPN, Centre for Interdisciplinary Research, Faculty of Biotechnology and Environmental Sciences, John Paul II Catholic University of Lublin, ul. Konstantynów 1J, 20-708 Lublin, Poland; marskow@kul.pl; 4 Department of Pharmacognosy, Faculty of Pharmacy, Medical University of Bialystok, ul. Mickiewicza 2a, 15-230 Białystok, Poland; michal.tomczyk@umb.edu.pl

**Keywords:** *Laetiporus sulphureus*, prebiotic, α-(1→3)-glucooligosaccharides, α-(1→3)-glucan, fruiting bodies

## Abstract

Increasing knowledge of the role of the intestinal microbiome in human health and well-being has resulted in increased interest in prebiotics, mainly oligosaccharides of various origins. To date, there are no reports in the literature on the prebiotic properties of oligosaccharides produced by the hydrolysis of pure fungal α-(1→3)-glucan. The aim of this study was to prepare α-(1→3)-glucooligosaccharides (α-(1→3)-GOS) and to perform initial evaluation of their prebiotic potential. The oligosaccharides were obtained by acid hydrolysis of α-(1→3)-glucan isolated from the fruiting bodies of *Laetiporus sulphureus* and then, characterized by HPLC. Fermentation of α-(1→3)-GOS and reference prebiotics was compared in in vitro pure cultures of *Lactobacillus*, *Bifidobacterium*, and enteric bacterial strains. A mixture of α-(1→3)-GOS, notably with a degree of polymerization of 2 to 9, was obtained. The hydrolysate was utilized for growth by most of the *Lactobacillus* strains tested and showed a strong bifidogenic effect, but did not promote the growth of *Escherichia coli* and *Enterococcus faecalis*. α-(1→3)-GOS proved to be effective in the selective stimulation of beneficial bacteria and can be further tested to determine their prebiotic functionality.

## 1. Introduction

In recent decades, the pivotal role of the human gut microbiome in health or disease state has been proven. The complex symbiotic balance of the microbiome can be disrupted by acute (e.g., antibiotic treatment) or chronic (e.g., some diseases) circumstances [1]. The resulting dysbiosis can be associated with many diseases such as inflammatory bowel disease, irritable bowel syndrome, metabolic syndrome, obesity, allergy, asthma, cardiovascular disease, and other conditions [2,3]. Some studies have shown that the composition of the gut microbiota can be manipulated through diet [4]. This is the case of supplementation of food with non-digestible carbohydrates or prebiotics, which, according to the classic definition proposed by Gibson and Roberfroid [5], are able to “beneficially affect the host by selectively stimulating the growth and/or activity of one or a limited number of bacteria in the colon, which can improve the host health”. Prebiotics have been shown to modulate the host immune system, play a role in regulating mineral and lipid metabolism, protect against colon cancer, and cardiovascular disease and metabolic syndrome [6,7].

Prebiotics include various polysaccharides (e.g., inulin), but also a large group of oligosaccharides containing from two to several monosaccharide units. The latter are starch derivatives, including maltooligosaccharides, isomaltooligosaccharides, nigero-oligosaccharides, cyclodextrins, trehalose, and gentiooligosaccharides; sucrose derivatives including fructooligosaccharides (FOS), isomaltulose, raffinose, and stachyose; lactose derivatives, including galactooligosaccharides, lactulose, and lactitol; and many others such as chitin-/chitosanoligosaccharides, mannooligosaccharides, xylooligosaccharides, and agarooligosaccharides [8,9]. Oligosaccharides can be obtained by extraction from natural sources, by hydrolysis of polysaccharides, or by synthesis from disaccharides [10,11]. Well-recognized oligosaccharides, fructooligosaccharides, and galactooligosaccharides, fulfill all the criteria for the classification of prebiotics [12,13]. However, new oligosaccharide candidates for the name “prebiotic” are still being extensively investigated [8,14].

To date, there are no literature reports on the prebiotic properties of oligosaccharides containing mainly α-(1→3)-glucosidic linkages and produced by the hydrolysis of pure α-(1→3)-glucans. Recently, α-(1→3)-glucooligosaccharides (α-(1→3)-GOS) obtained from α-(1→3)-glucan isolated from polypore fungus *Fomitopsis betulina* fruiting bodies have been characterized and their antiproliferative and pro-apoptotic properties against colon cancer, but not against normal epithelial colon cells, have been shown [15]. In the present study, we prepared oligosaccharides by acid hydrolysis of α-(1→3)-glucan isolated from a commonly available source, i.e., fruiting bodies of *Laetiporus sulphureus*. The in vitro growth response of *Lactobacillus*, *Bifidobacterium*, and enteric strains to α-(1→3)-glucooligosaccharides was evaluated as a measure of the prebiotic potential of the oligosaccharides.

## 2. Results and Discussion

Oligosaccharide prebiotics play an important role in regulating the diversity of human gut microbiota. They are widely used as functional additives to different foodstuffs, dietary supplements, and probiotic preparations. For this reason, new, easily available, and relatively cheap sources of these compounds with different activity to stimulate beneficial intestinal microbiome are still being sought. The hydrolysis of polysaccharides is a method for the production of potential prebiotic oligosaccharides [16]. In our study, cell wall α-(1→3)-glucan isolated from *L. sulphureus*, i.e., a *Polyporaceae* fungus widespread in the northern hemisphere, was the source of the oligosaccharides. The large fruiting bodies of *L. sulphureus* are one of the richest sources of glucan [17]. Fungal α-(1→3)-glucans are linear polymers of glucose linked with α-(1→3)-glucosidic bonds. They dissolve in alkali but not in water, which limits but does not exclude their biological activity [18]. Soluble, carboxymethylated, sulfated, or phosphated derivatives of α-(1→3)-glucan exhibit mainly antitumor activity [19,20,21].

**Figure 1:**
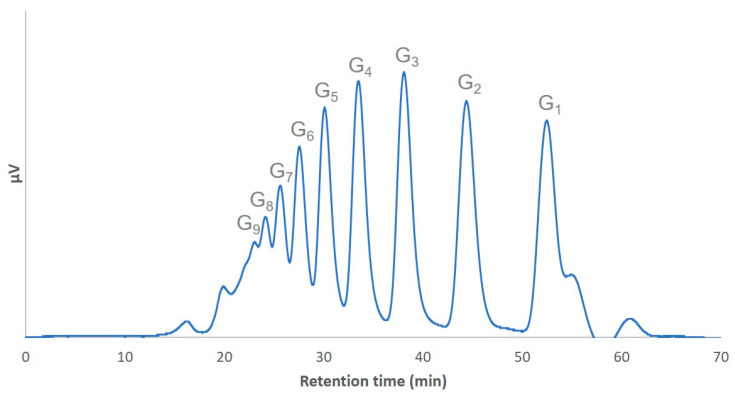
HPLC analysis of α-(1→3)-glucooligosaccharides obtained by partial acid hydrolysis of *Laetiporus sulphureus* α-(1→3)-glucan. In the abbreviation Gn, “n” represents the degree of polymerization.

The acid hydrolysis of the α-glucan yielded a mixture of α-(1→3)-glucooligosaccharides. The oligosaccharide profile in the mixture was analyzed by HPLC. The hydrolysate contained oligosaccharides (85.6%), especially with a degree of polymerization of 2 to 9 (Figure 1), including 17.5% dimers (nigerose, *O*-α-d-glucopyranosyl-(1→3)-d-glucopyranose), 16.8% trimers (nigerotriose, *O*-α-d-glucopyranosyl-(1→3)-*O*-α-d-glucopyranosyl-(1→3)-d-glucopyranose), 14.8% tetramers (nigerotetraose, *O*-α-d-glucopyranosyl-(1→3)-*O*-α-d-glucopyranosyl-(1→3)-*O*-α-d-glucopyranosyl-(1→3)-d-glucopyranose), and 12% pentamers (nigeropentaose, *O*-α-d-glucopyranosyl-(1→3)-*O*-α-d-glucopyranosyl-(1→3)-*O*-α-d-glucopyranosyl-(1→3)-*O*-α-d-glucopyranosyl-(1→3)-d-glucopyranose). Glucose accounted for 14.4% of the hydrolysate.

α-(1→3)-Glucooligosaccharides are usually identified with nigerooligosaccharides (NOS) as glucose oligomers containing one or more α-(1→3)-glucosidic linkages and having a degree of polymerization of 2 to 10. Shimamura et al. obtained nigerose and its homologous oligomers via partial hydrolysis of α-(1→3)-glucan (mutan) synthesized by a recombinant *Escherichia coli* strain containing a gtfI gene of *Streptococcus downei* MFe28 [22]. In addition to (1→3) linkages, NOS can contain (1→1), (1→2), (1→4), or (1→6) bonds in varying proportions. At the same time, the term “nigerooligosaccharides” is defined in the literature differently and can therefore be misleading. The glucooligosaccharides obtained in the present study contained mainly α-(1→3)-glucosidic linkages, whereas the name “NOS” usually refers to a mixture of nigerose, nigerosyl glucose, and nigerosyl maltose [23] or to a product of the hydrolysis of polysaccharides such as nigeran, i.e., an unbranched glucan consisting of alternating α-(1→4) and α-(1→3) linkages, or elsinan, comprising maltotriose and maltotetraose residues joined by α-(1→3)-linkages [24]. Goffin et al. proposed the inclusion of oligosaccharides understood in this way within the definition of isomaltooligosaccharides (IMO) due to their occurrence in IMO syrup, the presence of (1→6)-α-bonds, and a similar method for preparation from maltose via the transglucosidase activity of microbial α-glucosidases [25].

**Figure 2:**
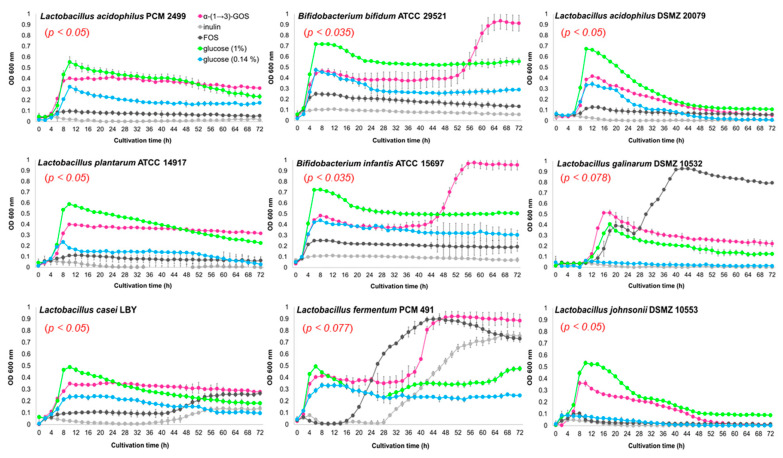
Growth curves of *Lactobacillus* and *Bifidobacterium* strains in basal medium supplemented with α-(1→3)-glucooligosaccharides (α-(1→3)-GOS), fructooligosaccharides (FOS), inulin, and glucose. The number of bacteria is reported as a change in optical density. The results are given as means ± SD of triplicate samples.

**Figure 3:**
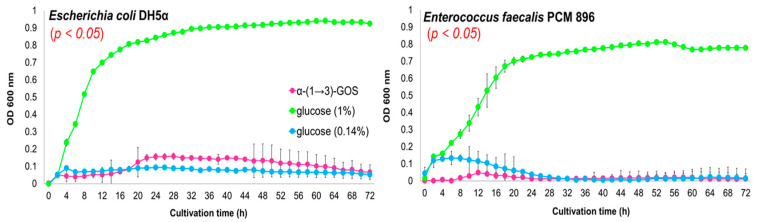
Growth curves of *Escherichia coli* DH5α and *Enterococcus faecalis* PCM 896 in basal medium supplemented with α-(1→3)-GOS and glucose. The number of bacteria is reported as a change in optical density. The results are given as means ± SD of triplicate samples.

In addition to the digestive resistance in the upper gastrointestinal tract and biological activity, the standard requirement for a carbohydrate to be regarded as prebiotic is the selective stimulation of the growth and/or activity of a limited number of gut microorganisms described as beneficial, and suppression of the growth of potentially detrimental or pathogenic ones [26]. For this reason, the in vitro fermentability of α-glucan hydrolysates by potential probiotic and enteric bacteria was investigated and compared to reference prebiotics, i.e., FOS and inulin. Considering bacterial growth in the control medium supplemented with 0.14% glucose (an amount reflecting the concentration of glucose in the medium with 1% of α-(1→3)-GOS) and comparing the growth in media with 1% glucose, α-(1→3)-GOS, or FOS/inulin, it was demonstrated that α-(1→3)-GOS were fermented by the lactobacilli and bifidobacteria tested (Figure 2). In addition, it was shown that α-(1→3)-GOS did not promote the growth of potentially pathogenic *E. coli* DH5α and *Enterococcus faecalis* PCM 896 (Figure 3). These non-probiotic enteric bacteria have been used as a simplified model of intestinal bacteria due to their broad use and isolation from the human gut [27,28,29]. Different growth phenotypes with α-(1→3)-GOS were observed for the various strains of *Lactobacillus* and *Bifidobacterium*. *Lactobacillus casei* LBY, *L. plantarum* ATCC 14917, and *L. acidophilus* PCM 2499 produced the highest cell density (OD_600_ of 0.35–0.40 with α-(1→3)-GOS vs. 0.24–0.32 with 0.14% glucose) after 10 h of culture. Next, they entered the stationary growth phase, which continued until the end of the culture. The final OD_600_ values with α-(1→3)-GOS were still high (0.3) in contrast to the values with glucose (0.18–0.03) (Figure 2). Biphasic growth curves were observed in the case of *Bifidobacterium longum* subsp. *infantis* ATCC 15697, *Bifidobacterium bifidum* ATCC 29521, and *Lactobacillus fermentum* PCM 491. Until 36–48 h of culture, the strains grew in a similar way to those described above, and then, they began to utilize another carbon source (possibly higher α-(1→3)-glucooligosaccharides), entered the second log phase, achieved very high cell density (max. OD_600_ > 0.9 with α-(1→3)-GOS vs. 0.28–0.45 with glucose), and then, reached a plateau.

In turn, despite their very poor growth in the medium with glucose, *L. johnsonii* DSMZ 10553 and *L. gallinarum* DSMZ 10532 reached OD_600_ of 0.36 and 0.51, respectively, with α-(1→3)-GOS and then, entered the decline phase. *L. acidophilus* DSMZ 20079 was the only strain whose growth with α-(1→3)-GOS resulted only from the use of glucose present in the hydrolysate. Interestingly, for most strains (except for *L. fermentum*, *L. gallinarum*, and *L. casei*), there was no significant effect of the reference prebiotics on the growth of the bacteria tested. *L. fermentum* reached a final OD_600_ of 0.88 with the hydrolysate of α-glucan, 0.73 with FOS, and 0.76 with inulin. These results suggest that this strain, just like *L. casei*, can utilize the hydrolysate at the same appreciable level as the reference prebiotics, FOS and inulin, or only FOS, respectively. Based on the growth curve parameters (max specific growth rate, lag time, etc.), which were determined using a Python script (Tables S1 and S2), we can confirm that the R^2^ value assigned to the α-(1→3)-GOS clearly indicates that the fit of the statistical analysis was very high.

Synytsya et al. found that α-(1→3)-glucan isolated from the cell wall of two *Pleurotus* species alone can act as a nutrient for probiotic bifidobacteria and lactobacilli [18]. However, in contrast to the α-(1→3)-GOS tested in our study, its properties, such as insolubility in water, limit the possibilities of its use as a functional additive to foodstuffs. The prebiotic properties of many oligosaccharides are widely studied and are well known, whereas in the group of α-(1→3)-glucooligosaccharides, the prebiotic activity of only certain types of nigerooligosaccharides has been documented. Nigerooligosaccharides have a somewhat similar structure to α-(1→3)-GOS but they are produced enzymatically, e.g., from maltose using *Acremonium* sp. [30]. They are widely used in Japan as Food for Specified Health Use (FOSHU), for instance as a syrup for improving the taste and color of many kinds of foods and beverages [31]. They have been found effective in suppression of the reactivity of the superoxide ion and in the enhancement of immunity [32,33,34,35], while nigerose, present in very small quantities in traditional low alcohol Japanese drinks such as sake and amazake, promotes the growth of *Bifidobacterium* species [30]. Indigestible oligosaccharides, e.g., cyclic nigerosylnigerose with four d-glucopyranosyl residues linked by alternating α-(1→3)- and α-(1→6)-glucosidic linkages, also have prebiotic activity. In in vivo experiments, they were able to beneficially modify the intestinal environment of murine microbiota and activate the mucosal immune system [36].

## 3. Materials and Methods

### 3.1. Materials

The fruiting bodies of *Laetiporus sulphureus* (Bull.: Fr.) Murrill were harvested from various host trees in Lublin, Poland. Inulin Raftiline HP and fructooligosaccharides (FOS) Raftilose P95 were purchased from the Orafti Group (Tienen, Belgium). De Man, Rogosa, and Sharpe medium (MRS) was obtained from BTL (Łódź, Poland), and Tryptic Soy Broth (TSB) was supplied by Difco Laboratories (Detroit, MI, USA). Amberlite MB3 was purchased from Merck (Darmstadt, Germany). All other chemicals were obtained from Sigma-Aldrich Chemical Co. (St. Louis, MO, USA) unless otherwise stated.

The following bacteria were used: *Lactobacillus acidophilus* DSMZ 20079 (German Collection of Microorganisms and Cell Cultures, Braunschweig, Germany) and PCM 2499 (Polish Collection of Microorganisms, Polish Academy of Sciences, Institute of Immunology and Experimental Therapy, Wrocław, Poland), *L. plantarum* ATCC 14917 (American Type Culture Collection, Manassas, VA, USA), *L. fermentum* PCM 491, *L. casei* LBY (Division of Food Science Institute of Animal Reproduction and Food Research of the Polish Academy of Sciences, Olsztyn, Poland), *L. gallinarum* DSMZ 10532, *L. johnsonii* DSMZ 10533, *Bifidobacterium longum* subsp. *infantis* ATCC 15697, *B. bifidum* ATCC 29521, *Escherichia coli* DH5α, and *Enterococcus faecalis* PCM 896.

### 3.2. Preparation and Analysis of the α-(1→3)-Glucooligosaccharide Preparation

An alkali-soluble α-(1→3)-glucan was prepared from the cell wall material isolated from the fruiting bodies of *L. sulphureus* according to Wiater et al. [37]. Crude oligosaccharides were obtained by partial hydrolysis of the glucan in 0.1 M H_2_SO_4_ for 1 h at 100 °C. The residues were removed by centrifugation (10 min, 12,000 rpm) and the supernatant was neutralized with CaCO_3_. After re-centrifugation, the soluble fraction of the hydrolysate was desalted with Amberlite MB3. The desalted solution was concentrated at 40 °C using a rotary evaporator under vacuum and freeze-dried. The preparation containing the mixture of α-(1→3)-glucooligosaccharides was analyzed by HPLC using the chromatographic system Prominence LC-20A (Shimadzu, Kyoto, Japan) connected to a refractive index detector (RID-10). The mobile phase (Milli-Q water) was run at a flow rate of 0.25 mL/min at 40 °C through a Rezex RSO-Oligosaccharide Ag^+^ column (1 cm × 20 cm, Phenomenex, CA, USA). The column was calibrated using the following sugars: maltooligosaccharide standard (light corn syrup) with a degree of polymerization from 1 to 14, nigerotetraose, nigerose, and glucose (Sigma, St. Louis, MO, USA).

### 3.3. In Vitro Effects of α-(1→3)-GOS on Bacterial Growth

The growth of the selected bacteria on the substances tested was determined using an Automated Microbiology Growth Curve Analysis System, Bioscreen C (LabSystem, Finland) in dedicated 100-microwell plates. Bacterial strains stored at −80 °C in MRS/TSB broth containing 20% glycerol were sub-cultured (overnight, in anaerobic conditions, 37 °C) in the MRS medium supplemented with 0.05% l-cysteine-HCl (*Lactobacillus* and *Bifidobacterium*) and in TSB (*Escherichia* and *Enterococcus*) prior to use. The harvested cells were washed three times in phosphate-buffered saline and standardized to OD_600_ of 1 with a fresh carbohydrate-free MRS or TSB medium. The wells of the honeycomb plates were filled with 350 μL of carbohydrate-free MRS or TSB medium supplemented with filter-sterilized α-(1→3)-GOS or reference prebiotics (inulin/FOS) to a final concentration of 1% (*w*/*v*). Media with glucose at a concentration of 0.14% (an amount corresponding to the concentration of glucose in the 1% α-(1→3)-GOS preparation) and 1% (*w*/*v*) were used as controls, and additionally, carbohydrate-free media served as negative controls. The media were then inoculated with 50 μL of standard bacterial suspensions and cultured anaerobically (under a layer of mineral oil) for 72 h at 37 °C. The growth rate of each strain was monitored by measuring the optical density (OD_600_) every two hours. Each carbohydrate was tested in a minimum of 3 independent replicates.

### 3.4. Statistical Analysis

Analyses were performed in triplicate with three biological replicates for each condition. Data were subjected to one-way ANOVA; pair-comparison of treatment means was achieved by Tukey’s procedure at *p* < 0.05, using the statistical software, Statistica for 245 Windows (Statistica 7.0 ver Windows). Growth curve parameters (max specific growth rate, lag time, doubling time, etc.) were determined using a Python script according to Hoeflinger et al. [38].

## 4. Conclusions

In this preliminary study, a novel, water-soluble α-(1→3)-glucooligosaccharides group containing 2 to 9 sugar units was obtained by acid hydrolysis of α-(1→3)-glucan isolated from the fruiting bodies of polypore fungus *Laetiporus sulphureus*, and their prebiotic potential was evaluated. Based on OD measurements, the in vitro fermentation of this oligosaccharide mixture promoted the growth of tested reference probiotic lactobacilli and bifidobacterial strains, but not potentially pathogenic *Escherichia coli* and *Enterococcus faecalis*. The results obtained indicate that α-(1→3)-GOS proved to be effective in the selective stimulation of beneficial bacteria and can be further tested to determine their prebiotic features. In turn, finding and using a rich, inexpensive, safe for humans, and renewable source of fungal α-(1→3)-glucan to prepare α-(1→3)-oligosaccharides gives promising prospects for the production of these new prebiotic compounds on a larger scale. Thus, further experiments are currently in progress to determine α-(1→3)-GOS digestibility and assimilation, the end products of fermentation, such as short-chain fatty acids (SCFA), and quantitative and qualitative changes in the composition of the intestinal microbiota of mice fed with our probiotic α-(1→3)-GOS.

## Supplementary Materials

The following are available online. Table S1: Kinetic parameters of *Lactobacillus* and *Bifidobacterium* growth on different carbon source by Python analysis; Table S2: Kinetic parameters of enteric bacterial strains growth on different carbon source by Python analysis.
