# Supplementary material for: Prebiotic Potential of Oligosaccharides Obtained by Acid Hydrolysis of α-(1→3)-Glucan from Laetiporus sulphureus: A Pilot Study
Source: Molecules. 2020 Nov 26;25(23):5542. doi: 10.3390/molecules25235542 (PMC7728339; doi:10.3390/molecules25235542)
Supplement: Supplementary file 1 [file molecules-25-05542-s001.pdf]

Supplementary Materials

Acid Hydrolysis of α-(1→3)-Glucan from *Laetiporus sulphureus*: A Pilot Study

Adrian Wiater [1,*], Adam Waśko [2,*], Paulina Adamczyk [1], Klaudia Gustaw [2], Małgorzata Pleszczyńska [1], Kamila Wlizło [1], Marcin Skowronek [3], Michał Tomczyk [4], and Janusz Szczodrak [1]

[1] Department of Industrial and Environmental Microbiology, Institute of Biological Science, Maria Curie-Skłodowska University, Akademicka 19, 20-033 Lublin, Poland; adrianw2@poczta.umcs.lublin.pl (A.Wi.); paulinapolak2501@gmail.com (P.A.); m.pleszczynska@poczta.umcs.lublin.pl (M.P.); kamila.wlizlo@poczta.umcs.lublin.pl (K.W.); szczo@poczta.umcs.lublin.pl (J.S.)

[2] Department of Biotechnology, Human Nutrition and Food Commodity Science, University of Life Sciences in Lublin, Skromna 8, 20-704 Lublin, Poland; awasko1@tlen.pl (A.Wa.); kowalikklaudia2105@gmail.com (K.G.)

[3] Laboratory of Biocontrol, Application and Production of EPN, Centre for Interdisciplinary Research, Faculty of Biotechnology and Environmental Sciences, John Paul II Catholic University of Lublin, ul. Konstantynów 1J, 20-708 Lublin, Poland; marskow@kul.pl (M.S.)

[4] Department of Pharmacognosy, Faculty of Pharmacy, Medical University of Bialystok, ul. Mickiewicza 2a, 15-230 Białystok, Poland; michal.tomczyk@umb.edu.pl (M.T.)

* Correspondence: adrianw2@poczta.umcs.lublin.pl (A.Wi.); awasko1@tlen.pl (A.Wa.)

Table S1. Kinetic parameters of *Lactobacillus* and *Bifidobacterium* growth on different carbon source by Python analysis.

Strain	Carbon Source	Lag Time (hours)	Max Specific Growth Rate (1/hours)	Doubling Time (hours)	Max OD	Max OD (Median Filtered Data)	Min OD	Min OD (Median Filtered Data)	Delta OD (Median Filtered Data)	R^2
L. johnsonii DSMZ 10533	FOS	4.99449	0.177485	3.905391	1.7417	1.739417	0.02475	0.03475	1.704667	0.99875
	Inulin	5.93918	0.167702	4.133218	1.5183	1.517636	0.01556	0.053636	1.464	0.99656
	α-(1→3)-GOS	1.90849	0.050426	13.74575	0.7166	0.713697	0.12	0.12	0.593697	0.99740
	Glucose 1%	4.54453	0.163271	4.245388	1.6084	1.596773	0.0326	0.0326	1.564173	0.99845
	Glucose 0.14%	1.0E-06	0.053053	13.06519	0.6222	0.616286	0.0236	0.0236	0.592686	0.97678
L. casei LBY	FOS	44.1322	0.014292	48.49917	0.2666	0.266667	0.00496	0.004967	0.2617	0.94425
	Inulin				0.1375	0.137167	0.00563	0.005633	0.131533	
	α-(1→3)-GOS	2.17316	0.048035	14.43013	0.358	0.3515	0.0016	0.0016	0.3499	0.99539
	Glucose 1%	2.76345	0.033713	20.56004	0.2412	0.2407	0.00636	0.006367	0.234333	0.99005
	Glucose 0.14%				0.0767	0.0714	0.0094	0.0094	0.062	
L. galinarium DSMZ 10532	FOS	13.87790	0.03306	20.96644	0.9293	0.929	0.0142	0.0142	0.9148	0.97439
	Inulin				0.0426	0.041667	0.00013	0.0166	0.025067	
	α-(1→3)-GOS	12.45017	0.13651	5.077624	0.5131	0.512333	0.0139	0.0139	0.498433	0.99881
	Glucose 1%	12.52688	0.067282	10.30214	0.407	0.343333	0.0153	0.0153	0.328033	0.99694
	Glucose 0.14%				0.0627	0.0564	0.00066	0.011667	0.044733	
L. plantarum ATCC 14917	FOS				0.1116	0.111667	0.01413	0.014133	0.097533	
	Inulin				0.0593	0.057333	-0.007	0.017833	0.0395	
	α-(1→3)-GOS	6.056847	0.100751	6.87982	0.4	0.395167	0.01306	0.013067	0.3821	0.997529
	Glucose 1%				0.2355	0.191133	0.01433	0.014333	0.1768	
	Glucose 0.14%				0.0770	0.077007	0.02593	0.025933	0.051073	

B. infantis ATCC 15697	FOS				0.252	0.251333	0.04666	0.046667	0.204667	
	Inulin				0.1116	0.110667	0.068	0.070333	0.040333	
	α-(1→3)-GOS	46.09435	0.069354	9.994277	0.9765	0.9685	0.036	0.036	0.9325	0.919588
	Glucose 1%	2.829885	0.138591	5.001388	0.4387	0.422333	0.051	0.051	0.371333	0.756334
	Glucose 0.14%				0.2124	0.21144	0.05377	0.06344	0.148	
L. acidophilus DSMZ 20079	FOS				0.128	0.126333	0.04933	0.05	0.076333	
	Inulin				0.05	0.049667	0.00066	0.035667	0.014	
	α-(1→3)-GOS	8.451172	0.179685	3.857568	0.4175	0.4015	0.03266	0.032667	0.368833	0.999801
	Glucose 1%	8.074748	0.130782	5.300024	0.3439	0.333667	0.00766	0.051333	0.282333	0.998417
	Glucose 0.14%				0.1137	0.11271	0.00993	0.023895	0.088815	
L. fermentum PCM 491	FOS	18.59824	0.055403	12.51089	0.9	0.898	0.00666	0.007333	0.890667	0.99543
	Inulin	32.21000	0.032723	21.18248	0.7625	0.758833	0.00333	0.0085	0.750333	0.99697
	α-(1→3)-GOS	38.22799	0.091396	7.584034	0.922	0.92	0.029	0.029	0.891	0.92919
	Glucose 1%	0.000001	0.035912	19.30139	0.3418	0.324	0.043	0.043	0.281	0.91041
	Glucose 0.14%				0.0854	0.082107	0.00422	0.01256	0.069547	
L. acidophilus PCM 2499	FOS				0.097	0.096	0.03633	0.042667	0.053333	
	Inulin				0.049	0.044667	0	0.037333	0.007333	
	α-(1→3)-GOS	5.296628	0.107113	6.471185	0.414	0.4045	0.0295	0.0295	0.375	0.9988
	Glucose 1%	6.608075	0.07989	8.676292	0.3236	0.299333	0.01466	0.018	0.281333	0.998391
	Glucose 0.14%				0.074	0.074	0.009	0.074	0	
B. bifidum ATCC 29521	FOS				0.251	0.247333	0.055	0.055	0.192333	
	Inulin				0.1076	0.105667	0.05766	0.058333	0.047333	
	α-(1→3)-GOS	54.09968	0.06681	10.37484	0.9355	0.9245	0.038	0.038	0.8865	0.919025
	Glucose 1%	3.006118	0.143954	4.815073	0.4751	0.455197	0.02115	0.021159	0.434038	0.503862
	Glucose 0.14%				0.0333	0.032333	0.014	0.014	0.018333	

Table S2. Kinetic parameters of enteric bacterial strains growth on different carbon source by Python analysis.

Strain	Carbon Source	Lag Time (hours)	Max Specific Growth Rate (1/hours)	Doubling Time (hours)	Max OD	Max OD (Median Filtered Data)	Min OD	Min OD (Median Filtered Data)	Delta OD (Median Filtered Data)	R^2
E. coli DH5	α-(1→3)-GOS	1E-06			0.158833	0.156	0.0025	0.0025	0.1535	0.973473
	Glucose 1%	1E-06	0.068431	10.12908	0.941333	0.940333	0	0	0.940333	0.982145
	Glucose 0.14%	1E-06			0.094	0.094	0.004667	0.004667	0.089333	0.987652
E. faecalis PCM 896	α-(1→3)-GOS	1E-06			0.048667	0.041217	0.001233	0.001233	0.039983	0.984567
	Glucose 1%	1E-06	0.039592	17.50721	0.812833	0.812133	0.016033	0.016033	0.7961	0.992246
	Glucose 0.14%	1E-06			0.134	0.133	0.005667	0.0445	0.0885	0.967893
